# Fecal microbiota of horses with colitis and its association with laminitis and survival during hospitalization

**DOI:** 10.1111/jvim.16562

**Published:** 2022-10-21

**Authors:** Cosette Ayoub, Luis G. Arroyo, Jennifer L. MacNicol, David Renaud, J. Scott Weese, Diego E. Gomez

**Affiliations:** ^1^ Department of Clinical Studies, Ontario Veterinary College University of Guelph Guelph Ontario Canada; ^2^ Department of Animal Biosciences, Ontario Agricultural College University of Guelph Guelph Ontario Canada; ^3^ Department of Population Medicine, Ontario Veterinary College University of Guelph Guelph Ontario Canada; ^4^ Department of Pathobiology, Ontario Veterinary College University of Guelph Guelph Ontario Canada

**Keywords:** *Clostridium*, diarrhea, Enterobacteriaceae, *Lactobacillus*, *Streptococcus*

## Abstract

**Background:**

The association of microbiota with clinical outcomes and the taxa associated with colitis in horses remains generally unknown.

**Objectives:**

Describe the fecal microbiota of horses with colitis and investigate the association of the fecal microbiota with the development of laminitis and survival.

**Animals:**

Thirty‐six healthy and 55 colitis horses subdivided into laminitis (n = 15) and non‐laminitis (n = 39, 1 horse with chronic laminitis was removed from this comparison) and survivors (n = 27) and nonsurvivors (n = 28).

**Methods:**

Unmatched case‐control study. The Illumina MiSeq platform targeting the V4 region of the 16S ribosomal RNA gene was used to assess the microbiota.

**Results:**

The community membership (Jaccard index) and structure (Yue and Clayton index) were different (analysis of molecular variance [AMOVA]; *P* < .001) between healthy and colitis horses. The linear discriminant analysis effect size (LEfSe; linear discriminant analysis [LDA] >3; *P* < .05) and random forest analyses found Enterobacteriaceae, *Lactobacillus*, *Streptococcus*, and *Enterococcus* enriched in colitis horses, whereas *Treponema*, *Faecalibacterium*, Ruminococcaceae, and Lachnospiraceae were enriched in healthy horses. The community membership and structure of colitis horses with or without laminitis was (AMOVA; *P* > .05). Enterobacteriaceae, *Streptococcus*, and *Lactobacillus* were enriched in horses with laminitis (LDA > 3; *P* < .05)*.* The community membership (AMOVA; *P* = .008) of surviving and nonsurviving horses was different. Nonsurviving horses had an enrichment of Enterobacteriaceae, *Pseudomonas*, *Streptococcus*, and *Enterococcus* (LDA >3; *P* < .05).

**Conclusion and Clinical Importance:**

Differences in the microbiota of horses with colitis that survive or do not survive are minor and, similarly, the microbiota differences in horses with colitis that do or do not develop laminitis are minor.

**Abbreviations**
AMOVA: analysis of molecular variance
DMM: Dirichlet‐multinomial model
LDA: linear discriminatory analysis
LEfSe: linear discriminant analysis effect size
NNET: neural networks analysis
PCoA: principal coordinates analysis
RF: random forest
ROC: receiver operator curve
SIRS: systemic inflammatory response syndrome
SVM: support vector machine classifier

## 1 INTRODUCTION

Colitis is a major cause of morbidity and mortality in horses worldwide. Colitis is inflammation of a horse's colon and cecum resulting in watery diarrhea.^1^ Clinical pathological abnormalities can include tachycardia, fever, abnormal color of the mucous membranes, leukopenia, hypoproteinemia, and electrolyte abnormalities.^2^, ^3^ Known infectious agents in adult horses that cause colitis include *Salmonella* spp., *Clostridioides difficile*, *Clostridium perfringens*, *Neorickettsia risticii*, equine coronavirus, and small strongyles.^2^ Noninfectious causes consist of antimicrobial drugs associated diarrhea, sand impaction, dietary imbalances, neoplasia, and toxicities (eg, nonsteroidal antiinflammatory drugs).^1^ Regardless of the cause of diarrhea, alteration of the gastrointestinal microbiota (also known as dysbiosis) has been reported in horses.^4^, ^5^, ^6^ In healthy horses, the gastrointestinal microbiota is comprised of a high abundance of bacteria from the phylum Firmicutes and Bacteriodetes, with minor abundance of Fibrobacter and Proteobacteria.^4^ During diarrhea, the abundance of Firmicutes, Clostridia, Ruminococcaceae, and Lachnospiraceae decreases, whereas Enterobacteriaceae, *Lactobacillus*, and *Fusobacteria* increase.^4^, ^5^, ^6^ Three studies have reported changes in the microbiota of diarrheic horses, but included small sample sizes (6‐10 horses with diarrhea).^4^, ^5^, ^6^ Therefore, the impact on clinical outcome (e.g., survival, development of laminitis) and key bacterial groups associated with health and disease remain generally unknown.

The proportion of horses surviving colitis varies depending on the causative agent, geographic location, and the development of complications such as endotoxemia, sepsis, or laminitis.^7^, ^8^, ^9^, ^10^, ^11^, ^12^, ^13^ Horses with colitis that develop laminitis have an increased risk of mortality compared with those without laminitis.^14^ Laminitis has been associated with alteration of the gastrointestinal microbiota and absorption of microbial byproducts through permeable inflamed intestinal mucosa.^15^, ^16^ Several experimental studies have established a correlation between certain groups of bacteria (ie, *Lactobacillus* and *Streptococcus*) and the development of laminitis induced by administration of high concentrations of dietary carbohydrates (starch or oligofructose).^17^, ^18^, ^19^, ^20^ However, studies investigating whether similar microbial changes occur in horses with colitis that develop laminitis are lacking.

In critically ill human patients after trauma, burn, or spinal cord injury, an association between gastrointestinal microbiota, bacteremia, and mortality has been identified.^21^, ^22^, ^23^, ^24^, ^25^, ^26^, ^27^ Humans with severe systemic inflammatory response syndrome (SIRS) or bacteremia have fewer total obligate anaerobes, *Bifidobacterium* and *Lactobacillus*, which are predictive of mortality.^21^, ^28^, ^29^, ^30^ A lower fecal relative abundance of Firmicutes is reported in surviving critically ill humans, whereas, in nonsurvivors, a higher fecal relative abundance of Proteobacteria occurs.^29^, ^30^ These findings indicate that the alteration of the gastrointestinal microbiota can be a potential prognostic factor in critically ill animals. Our main objective was to describe the fecal microbiota of a large cohort of horses with colitis and to evaluate the changes in bacterial communities of colitis horses that develop laminitis. Our second objective was to investigate the fecal microbiota of surviving and nonsurviving horses with colitis.

## 2 MATERIALS AND METHODS

### 2.1 Ethic statement

This study was approved by the University of Guelph Animal Care Committee (AUP#4540).

### 2.2 Horses with colitis

Fecal samples from horses >1 year of age diagnosed with colitis at the Ontario Veterinary College Health Sciences Centre (OVC‐HSC) admitted between January 2014 and December 2019 were collected on admission and immediately stored at −80°C until processing. All fecal samples were collected via transrectal palpation by lubrication and disposable rectal sleeves or immediately after defecation during the initial physical examination and before any treatment. The type housing, diet, travel history, and treatment with gastroprotectants before admission were not consistently recorded for this group of horses.

Colitis was defined as a horse with acute onset diarrhea (<24 hours before admission) that had fever or leukopenia, or both. Horses were excluded if they were <1 year of age, had received antimicrobial drugs in the month before admission, had received PO medication or product (eg, di‐tri‐octahedral smectite, fecal transplantation, probiotics) for treatment of the current episode of acute diarrhea. Horses that received nonsteroidal anti‐inflammatory drugs before admission for the current episode of colitis were included in the study.

Horses with colitis (n = 55) were further divided into the following groups: nonsurvivors (n = 28/55; 51%) and survivors (n = 27/55; 49%) to hospital discharge and between horses that developed laminitis (C‐L; n = 15/54; 28%) during hospitalization and those that did not (C‐NL; n = 39/54; 72%). Surviving horses were defined as horses that were discharged from the hospital. One horse with chronic laminitis before admission was removed from the laminitis analysis. Laminitis was defined as lameness, increased digital pulses, positive response to hoof testers, and radiographic evidence of rotation or distal displacement of the third phalanx.

### 2.3 Healthy horses

Three groups of healthy horses were included in the control group (n = 36). Healthy horses were >1 year of age and considered healthy based on physical examination. Horses were excluded if they had a gastrointestinal disease within 3 months before sample collection or if they had received antimicrobial drugs or gastroprotectants in the previous 6 months. Fifteen client‐owned horses from the state of Florida, (H‐l), 11 healthy horses belonging to the teaching herd of the University of Florida's College of Veterinary Medicine Equine Research Program Shared Herd (TH‐l), and 10 healthy horses of the Ontario Veterinary College teaching herd (TH‐II) were used as healthy controls. The H‐l horses were fed hay (coastal Bermuda) and various types and amounts of grain, TH‐l horses were kept on pasture supplemented with coastal Bermuda hay and grain (1 pound per day), whereas TH‐II horses were kept in a free‐range stable and fed Timothy hay diet with no grain supplementation. The diets of all 3 groups of healthy horses were consistent during the 3 months before sampling. All fecal samples were collected from the rectum using lubrication and disposable rectal sleeves or from feces immediately after defecation. Samples were refrigerated and stored at −80°C within 1 hour after collection until processing. All samples from healthy horses were collected during the summer of 2019.

### 2.4 Sample size calculation

Sample size was calculated using a Dirichlet‐multinomial model (DMM) with an expected number of 20 000 sequence reads (per animal) available for comparison, and an alpha and power of 5% and 80%, respectively. The calculation provided a minimum of 15 horses per group (eg, healthy horses and horses with colitis).^31^

### 2.5 Sample processing

Once thawed, bacterial DNA was extracted from 200 mg fecal samples using the E.Z.N.A Stool DNA Kit (Omega Bio‐TEK, GA; Catalogue no 101319‐056) and performed according to the manufacturer's protocol. Then, the V4 region of the 16s rRNA gene was amplified using the modified primers 515‐F and 806‐R^32^ as previously described.^33^ The PCR products were purified using Mag Bind RXNPure Plus Beads (Omega Bio‐TEK, GA) according to the manufacturer's instructions. Sequence analysis was performed using Illumina MiSeq (Illumina RTA v1.17.28; MCS v2.2) for 250 cycles from each end at Guelph's Agriculture and Food Laboratory.

### 2.6 Bioinformatic and statistical analysis

The software Mothur (1.45.2) was used to complete the bioinformatic analysis following the standard operating procedure.^34^, ^35^ Sequences that passed quality control were identified using ribosomal database project classifier, clustered at the genus level (97% similarity), and binned into phylotypes. Sub‐sampling was performed based on the sample with the minimum number of reads. Good's coverages were evaluated to ensure sub‐sampling representation.^36^ The Chao‐1 (richness), inverse Simpson's (diversity), and Shannon's evenness (evenness) indices were used to assess the alpha diversity of the fecal microbiota. Comparison between healthy and colitis groups was performed using the Wilcoxon test and the healthy, laminitis, non‐laminitis, and healthy, and survival and nonsurvival comparisons were performed using the nonparametric Steel Dwass test for multiple comparisons (JMP 16, SAS Institute). Beta diversity was assessed using the Jaccard (community membership) and Yue and Clayton (community structure) indices.^37^, ^38^ Analysis of molecular variance (AMOVA) was used to investigate differences between groups. Clustering between groups was represented using principal coordinates analysis (PCoA) on Jaccard and Yue and Clayton indices. Assessment of the number of different meta‐communities into which the data could be clustered was completed using the Dirichlet multinomial mixtures (DMM) method.^39^ Relative abundances of the main phyla, classes, orders, families, and genera were calculated and compared between groups using the Wilcoxon test or the nonparametric Steel Dwass test for multiple comparisons. Benjamini and Hochberg's false discovery rate analysis^40^ was used to adjust *P* values for multiple comparisons (R! Core Team, 2013). Linear discriminant analysis effect size (LefSe) analysis was completed to determine the taxa enriched in the different groups based on a *P* < .05 and LDA score >3.5.^41^ Machine learning algorithms (i.e., random forests [RF], neural networks [NNET], LDA, and support vector machine classifier [SVMC]) were used to determine the ability of the fecal microbiota to differentiate groups (i.e., healthy vs colitis) and predict outcomes (ie, laminitis and survival), and to identify microbial taxa associated with each outcome.^42^ Initially, all sequences were grouped at genus level and zero and near‐zero variance predictors were removed from downstream analysis. Each machine learning algorithm was trained for each outcome. Classifiers were built using 100 repeats of 10‐fold cross‐validation. Random forests were built with 1001 trees, and all other model variables were optimized for area under the curve with the “caret” R package v. 6.0.86. Model performance was assessed and compared using the area under the receiver operator characteristics (ROC) curves.^42^ Variable importance was calculated for the top‐performing model to identify taxa associated with disease status. Analysis for the machine learning was completed using R v. 3.5.3.

## 3 RESULTS

### 3.1 Horses with colitis

Horses with colitis were between 1 and 26 years of age (median, 8 years) with 28 (51%) being mares, 26 (47%) geldings, and 1 (2%) a stallion. The breeds of horses were as follows: Thoroughbred (n = 12), Warmblood (n = 10), Quarter Horse (n = 6), Standardbred (n = 6), pony (n = 5), mixed breed (n = 2), Canadian Sport Horse (n = 2), Arabian (n = 2), Appaloosa (n = 2), Holsteiner (n = 1), Morgan (n = 1), Belgian (n = 1), Miniature Horse (n = 1), Paint (n = 1), Zangersheide (n = 1), Hanoverian (n = 1), and Friesian (n = 1). Data regarding admission physical examination, CBC, serum biochemical profile, and venous blood gas analysis of horses with colitis are presented in Table 1. Thirty‐nine of 55 (71%) horses with colitis were tested for *Salmonella* spp. using 1 fecal culture, 22 with 2 cultures, 9 with 3 cultures, 4 with 1 culture, and 5 with 1 culture. Fifteen (27%) horses with colitis were tested for *Neorickettsia risticii* using a PCR test performed using blood or feces or both. Eleven (7%) colitis horses were tested for *Clostridioides difficile* toxins A and B using an ELISA assay and 5 (9%) horses were tested for equine coronavirus using a PCR test of feces. Four horses tested positive for *Neorickettsia risticii*, 4 for *Clostridioides difficile* toxins A and B, 3 for *Salmonella* spp. on a culture of intestinal contents, and 1 for equine coronavirus. All 3 horses positive for *Salmonella* spp., 3/4 for *N. risticci*, and 3/4 for *C. difficile* died or were euthanized during hospitalization. Two of 3 horses diagnosed with *Salmonella* spp. and 2/4 horses positive for *N. risticci* developed laminitis. Of the 55 horses, 15 developed laminitis, 39 did not, and 1 horse was removed from the analysis because it had chronic laminitis. Twenty‐seven horses survived to hospitalization and 28 did not survive. Of the 28 nonsurvivors, 9 (32%) horses had laminitis related to colitis, whereas 6/27 (22%) survivors had laminitis (*P* = .5).

**TABLE 1:** Selected physical examination findings, complete blood cell count, serum biochemical profile, and venous blood gas analysis of 55 diarrheic horses admitted to a tertiary teaching hospital

Variable	Laminitis, n = 15	Non‐laminitis, n = 39	Survival, n = 27	Nonsurvival, n = 28	Reference ranges^3^
Attitude					
*Bright*	5 (30%)	12 (30%)	9 (33%)	8 (29%)	N/A
*Obtunded*	10 (70%)	28 (70%)	18 (67%)	20 (71%)	N/A
Colic	5 (36%)	13 (33%)	6 (22%)	12 (43%)	N/A
Laminitis	15 (100%)	0 (0%)	6/27 (22%)	7/28 (25%)	N/A
Temperature (Celsius)	38 [37‐39]	38 [36‐40]	38 [36‐40]	38 [36‐40]	37.2‐38.3
Heart rate (bpm)	58 [36‐88]	48 [36‐100]	44 [36‐78]	60 [40‐100]	28‐44
Resp. rate (rpm)	24 [12‐60]	24 [12‐60]	20 [12‐60]	24 [12‐60]	10‐24
PCV (%)	46 [33‐70]	45 [25‐73]	38 [25‐66]	51 [5‐73]	36‐44
WBC (×10^9^/L)	4 [1‐13]	5 [2‐14]	6 [2‐14]	4 [1‐14]	5.1‐11.0
Neutrophils (×10^9^/L)	1 [0.2‐12]	2 [0.05‐11]	3 [0.5‐12]	1 [0.05‐10]	2.8‐7.7
Bands (×10^9^/L)	0.2 [0.03‐0.8]	0.2 [0.02‐2]	0.2 [0.04‐10]	0.3 [0.02‐2]	< 0.2
pH	7.4 [7.3‐7.4]	7.4 [7.0‐7.5]	7.4 [7.2‐7.5]	7.4 [7.0‐7.5]	7.32‐7.44
HCO_3_^−^ (mmol/L)	25 [18‐29]	26 [15‐38]	27 [20‐31]	24 [15‐38]	24‐28
PvCO_2_ (mmHg)	40 [32‐60]	43 [30‐59]	45 [30‐59]	40 [32‐60]	38‐49
Na^+^ (mmol/L)	131[119‐141]	132 [118‐141]	133 [120‐141]	129 [118‐139]	136‐144
K^+^ (mmol/L)	3 [2‐5]	3 [1.3‐5]	3 [2‐4]	3 [1.3‐5]	3.1‐4.3
Cl^−^ (mmol/L)	98 [82‐112]	98 [78‐107]	100 [81‐112]	96 [78‐107]	95‐104
L‐Lactate (mmol/L)	3 [0.7‐7]	2 [0.4‐14]	2 [0.4‐10]	3 [1‐14]	0‐2
Creatinine (μmol/L)	131 [60‐378]	127 [71‐445]	120 [60‐349]	153 [108‐445]	80‐130
Total protein (g/L)	58 [38‐66]	56 [40‐92]	60 [42‐78]	52 [38‐92]	58‐75

*Note*: One horse had chronic laminitis before admission was removed from the laminitis analysis.

Abbreviations: bpm, beats per minute; PCV, packet cell volume; PvCO_2_, partial pressure of carbon dioxide; rpm, respiration per minute; WBC, white blood cells.

### 3.2 Healthy horses

Healthy horses consisted of 22 mares and 14 geldings, and the range of age was 4 to 23 years old. The following breeds were represented: Arabian (n = 1), Connemara (n = 1), Quarter Horse (n = 1), American Paint Horse (n = 2), Warmblood (n = 2), Appaloosa (n = 3), mixed breed (n = 4), Thoroughbred (n = 12), and Standardbred (n = 10).

### 3.3 Sequence analysis

Overall, 20 504 022 raw sequences were obtained, after filtering and cleaning 12 896 405 were available for analysis (mean, 141 718 ± 27 518; median, 139 464; range, 71 138‐222 987). Subsampling was performed at 70 000 sequences per sample. Subsampling was considered adequate based on a good coverage index value of 99.9%. In total, 28 phyla, 55 classes, 96 orders, 215 families, and 554 genera, respectively, were identified.

#### 3.3.1 Fecal microbiota of healthy horses and horses with colitis

**FIGURE 1:**
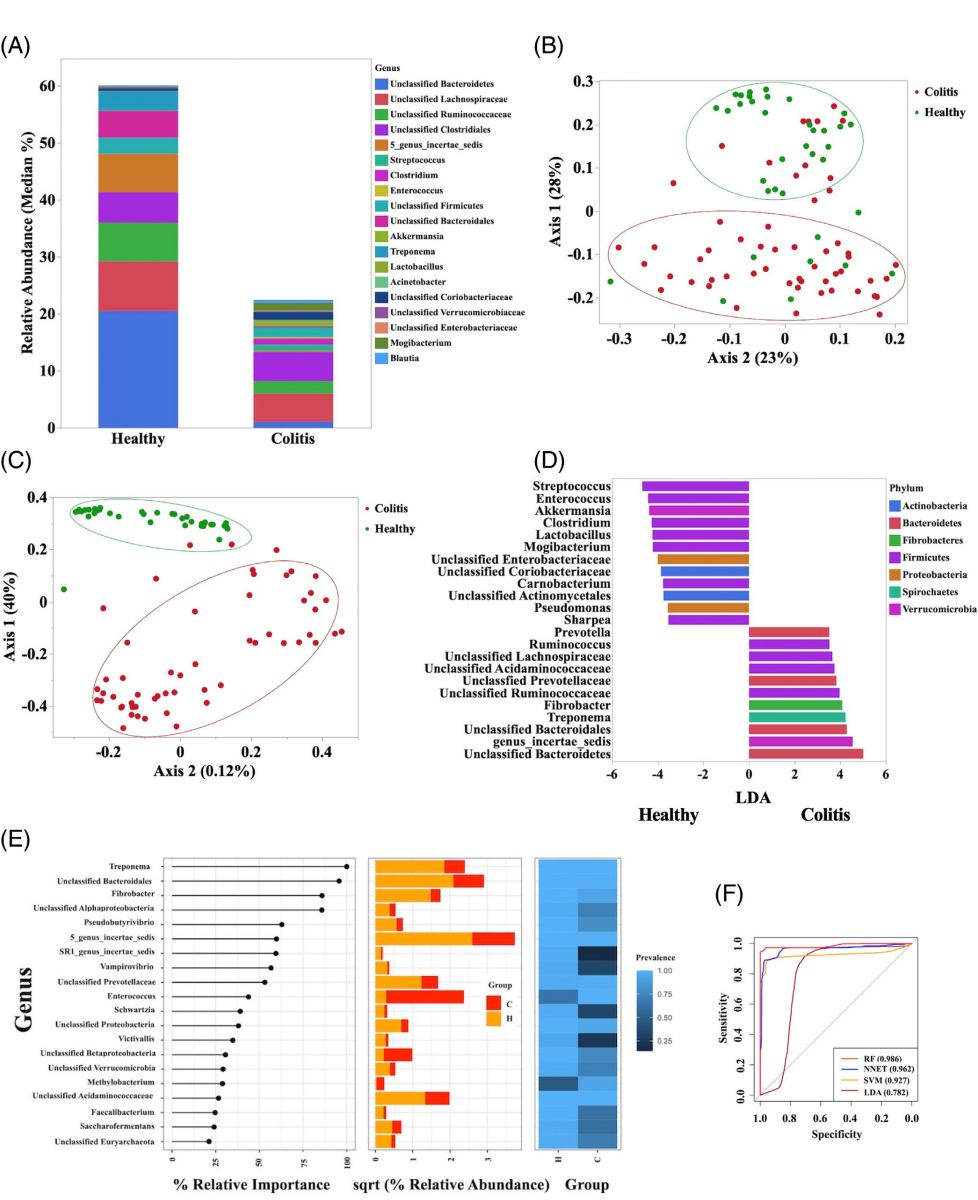
(A) Relative abundance (median) of the more abundant genera identified in healthy horses (n = 36) and horses with colitis (n = 55). (B) Principal coordinate analysis (PCoA) based on Jaccard index analysis of the bacterial 16S rRNA gene sequence data for fecal samples collected from healthy horses (n = 36) and horses with colitis (n = 55). (C) Principal coordinates analysis (PCoA) based on Yue and Clayton index analysis of the bacterial 16S rRNA gene sequence data for fecal samples collected from healthy horses (n = 36) and horses with colitis (n = 55). (D) Plot from LEfSe analysis indicating enriched taxa in fecal samples of healthy horses (n = 36) and horses with colitis (n = 55). Linear discriminant analysis (LDA) cut off >3.5 and *P* < .05. (E) Plot from random forest analysis showing the relative importance, abundance, and prevalence of taxa differentiating the fecal microbiota of healthy horses and horses with colitis. (F) Area under the receiving operator curves of the random forest (RF), neural network (NNET), support vector machine (SVM), and linear discriminate analysis (LDA) for prediction of diseases state (healthy vs colitis) based on the fecal microbiota profile

All 3 alpha diversity indices were similar between healthy horses and those with colitis (Table S1). Figure 1A depicts the relative abundance for the most abundant taxa identified in feces of healthy horses and horses with colitis. Significant differences in the community membership (Jaccard Index; AMOVA *P* < .001) and structure (Yue and Clayton Index; AMOVA *P* < .001) were detected between the healthy horses and horses with colitis. These differences were evident by the clustering of samples in the PCoA plots (Figure 1B,D). The LEfSe analysis indicated that samples from the healthy group were enriched with genera from the phyla Bacteroidetes, Firmicutes, Verrucomicrobia, Spirochaetes, and Fibrobacteres, whereas genera from the phyla Firmicutes, Proteobacteria, Actinobacteria, and Verrucomicrobia were enriched in horses with colitis (Figure 1C). In healthy horses, *Treponema*, *Ruminococcus*, *Prevotella* (*P* < .001), and an unclassified genus from the order Bacteroidales (*P* < .001), and families Lachnospiraceae (*P* = .03), Ruminococcaceae (*P* < .001), and Prevotellacease (*P* = .005) were enriched. The genera *Streptococcus* (*P* < .001), *Enterococcus* (*P* < .001), *Lactobacillus* (*P* = .003), and Enterobacteriaceae family (*P* < .001) were enriched in horses with colitis (Figure 1C). Using DMM, samples from healthy horses and horses with colitis were classified into 2 different metacommunities. Specifically, 88% (35/40) of samples in community 1 were from healthy horses and 12% (5/40) of samples were from horses with colitis, whereas 98% (50/51) of samples in community 2 were from horses with colitis and 2% (1/51) of samples were from healthy horses. The results of the random forest (RF) analysis showed that the genera *Treponema*, *Fibrobacter*, and an unclassified genus of the class *Alphaproteobacteria* and order *Bacterioidales* were more abundant in healthy horses than in horses with colitis. In horses with colitis, *Enterococcus*, *Methylobacterium*, and an unclassified genus from the class *Betaproteobacteria* were highly prevalent (Figure 1F). The RF analysis showed the highest predictive value (AUC ROC = 0.98). The machine learning models SVM and NNET also showed excellent ability to differentiate healthy horses from those with colitis based on the microbiota profile (AUC ROC > 0.90) (Figure 1F). The LDA analysis showed an acceptable predictive value to differentiate healthy from colitis horses (AUC ROC = 0.78).

#### 3.3.2 Laminitic vs non‐laminitic horses with colitis

**FIGURE 2:**
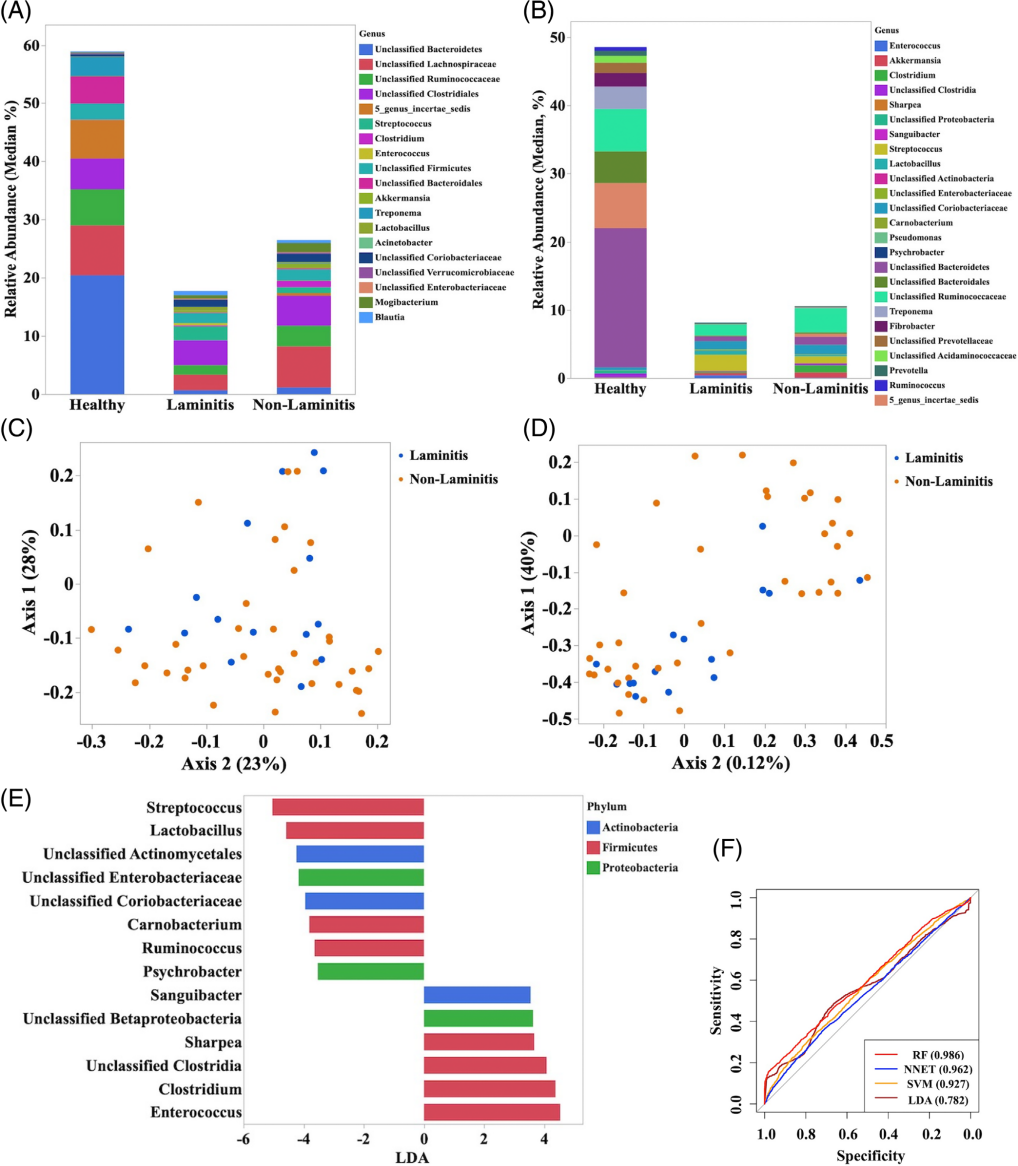
(A) Relative abundance (median) of the more abundant genera identified in horses with colitis with (n = 15) and without laminitis (n = 39) after removing one horse with chronic laminitis. (B) Principal coordinates analysis (PCoA) based on Jaccard index analysis of the bacterial 16S rRNA gene sequence data for fecal samples collected from horses with colitis with (n = 15) and without laminitis (n = 39). (C) Principal coordinates analysis (PCoA) based on Yue and Clayton index analysis of the bacterial 16S rRNA gene sequence data for fecal samples collected from horses with colitis with (n = 15) and without laminitis (n = 39). (D) Relative abundance analysis of the genera identified in the LEfSe analysis to be enriched in horses with colitis with (left, n = 15) and without (right, n = 39) laminitis. (E) Plot from LEfSe analysis indicating enriched taxa in fecal samples from horses with colitis with (n = 15) and without laminitis (n = 39). Linear discriminant analysis (LDA) cut off >3.5 and *P* < .05. (F) Area under the receiving operator curves of the random forest (RF), neural network (NNET), support vector machine (SVM), and linear discriminate analysis (LDA) for prediction of diseases state (laminitis vs non‐laminitis) based on the fecal microbiota profile

The alpha diversity indices were similar between laminitic and non‐laminitic horses (Table S2). The relative abundances of the most abundant taxa identified in feces of laminitic and non‐laminitic horses are presented in Figure 2A and Table S3. The community membership (Jaccard index; AMOVA *P* = .5) and structure (Yue and Clayton index; AMOVA *P* = .16) were not significantly different (Figure 2C,D). The LEfSe analysis (*P* < .05 and LDA > 3) of laminitis subgroups and healthy horses indicated that in laminitic horses, the genera *Streptococcus* and *Lactobacillus*, from phylum Firmicutes, and an unclassified genus of the family Enterobacteriaceae were enriched, whereas in the non‐laminitic group, *Enterococcus*, *Sharpea*, and *Clostridium* sensu stricto were enriched (Figure 2E,B). Using DMM, samples from laminitic and non‐laminitic horses with colitis were classified into a single metacommunity. The RF, SVM, LDA, and NNET analyses showed no discriminatory ability of the models to predict laminitis in horses with colitis based on microbiota profile (AUC ROC < 0.70; Figure 2F).

#### 3.3.3 Surviving vs nonsurviving horses with colitis

**FIGURE 3:**
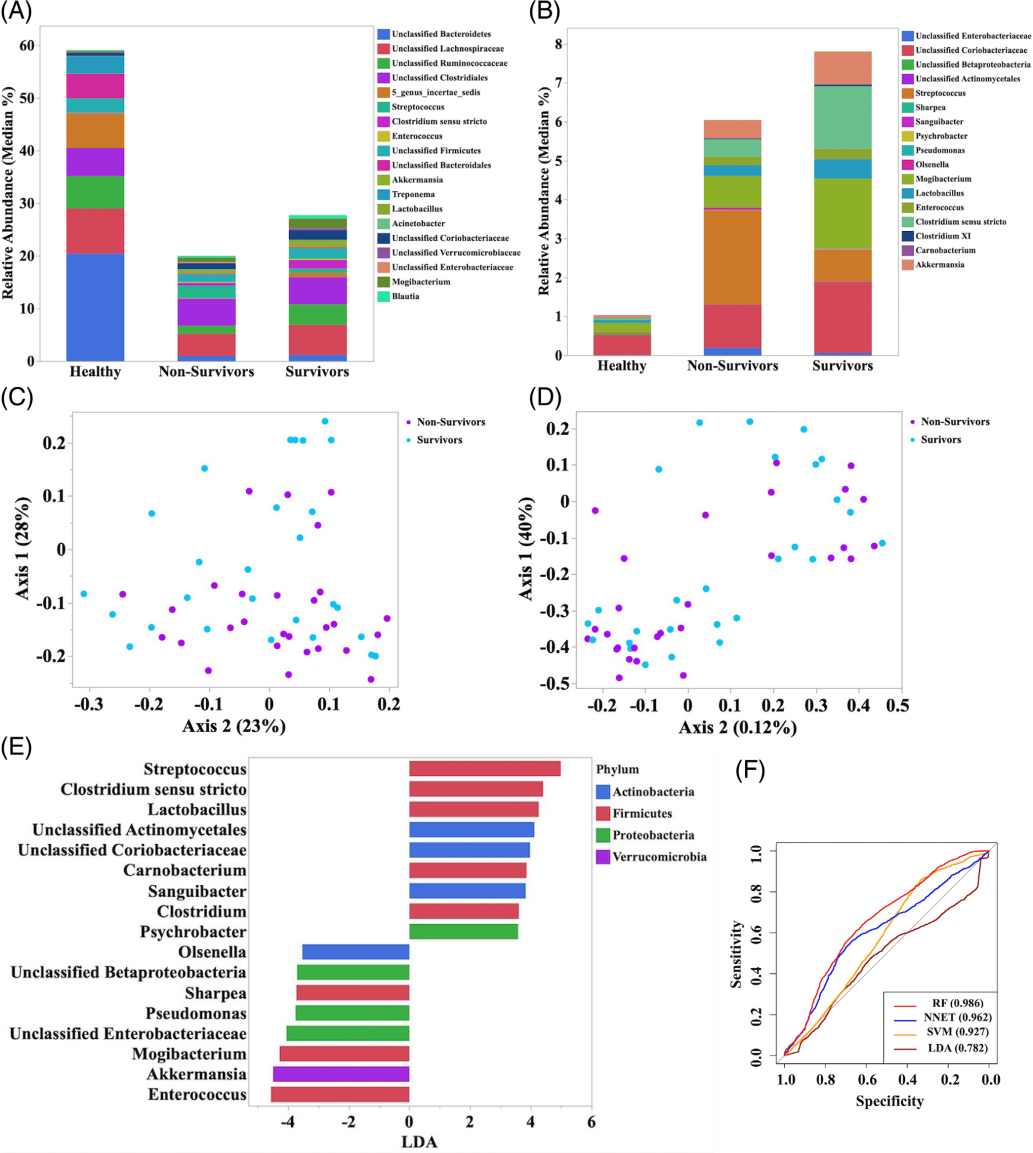
(A) Relative abundance (median) of the more abundant genera identified in surviving (n = 27) and nonsurviving (n = 28) horses with colitis. (B) Principal coordinates analysis (PCoA) based on Jaccard index analysis of the bacterial 16S rRNA gene sequence data for fecal samples collected from surviving (n = 27) and nonsurviving (n = 28) horses with colitis. (C) Principal coordinates analysis (PCoA) based on Yue and Clayton index analysis of the bacterial 16S rRNA gene sequence data for fecal samples collected from surviving (n = 27) and nonsurviving (n = 28) horses with colitis. (D) Relative abundance analysis of the genera identified in the LEfSe analysis to be enriched in surviving (n = 27) and nonsurviving (n = 28) horses with colitis. (E) Plot from LEfSe analysis indicating enriched taxa in fecal samples from surviving (n = 27) and nonsurviving (n = 28) horses with colitis. Linear discriminant analysis (LDA) cut off >3.5 and *P* < .05. (F) Area under the receiving operator curves of the random forest (RF), neural network (NNET), support vector machine (SVM), and linear discriminate analysis (LDA) for prediction of outcome (surviving versus nonsurviving) based on the fecal microbiota profile

No significant differences were identified in alpha diversity measurements between surviving and nonsurviving horses with colitis (Table S2). The relative abundance of the most abundant taxa identified in feces of survivors and nonsurvivors is presented in Figure 3A and Table S4. Community membership (Jaccard index; AMOVA *P* = .7) and structure (Yue and Clayton index; AMOVA *P* = .4) were not significantly different between survivors and nonsurvivors (Figure 3C,D). The LEfSe analysis (*P* < .05 and LDA > 3) indicated that the fecal microbiota of surviving horses was enriched with *Enterococcus*, *Mogibacterium*, *Sharpea*, *Pseudomonas*, Enterobacteriaceae, and an unclassified genus of the class Betaproteobacteria, whereas nonsurviving horses with colitis had enrichment of *Streptococcus*, *Clostridium* sensu stricto, *Lactobacillus*, *Actinomycetales*, and *Carnobacterium* (Figure 3B,E). Using DMM, samples from surviving and nonsurviving horses with colitis were classified into a single metacommunity. The RF, SVM, LDA, and NNET analysis showed no discriminatory capability of the models to predict survival of horses with colitis based on the admission fecal microbiota profile (AUC ROC < 0.70; Figure 3F).

A survival analysis was performed after removing horses that developed laminitis (n = 15) from the surviving (n = 6) and nonsurviving groups (n = 9). One surviving horse that had chronic laminitis at admission also was removed from this analysis. Statistical differences in the alpha diversity (richness, evenness and diversity; *P* > .05, for all comparisons) and beta diversity (Jaccard and Yue & Clayton indices) were not identified (AMOVA > .05 for both comparisons). The LEfSe analysis showed that *Lactobacillus* was no longer enriched in either group (Figure S1).

## 4 DISCUSSION

In our study, the differences in community membership and structure indicated that dysbiosis of the gastrointestinal tract was present in horses with colitis. This dysbiosis was associated with an enrichment in facultative anaerobes (ie, *Streptococcus*, *Lactobacillus*, *Enterococcus*, and Enterobacteriaceae) in diarrheic horses, whereas feces of healthy horses were enriched with obligate anaerobes such as *Treponema*, Ruminococcaceae, and Lachnospiraceae families. Alteration of the gastrointestinal microbiota has been reported in horses with diarrhea^4^, ^5^, ^6^ characterized by a decrease in the relative abundance of Firmicutes, Clostridia, Ruminococcaceae, and Lachnospiraceae. These results may suggest that a shift from obligate to facultative anaerobic bacteria, which is regarded as a marker of dysbiosis of the gastrointestinal tract in humans, calves, and horses,^43^, ^44^, ^45^ could occur in horses with colitis. Reasons for this shift are not completely understood, but during colitis intestinal contents are usually watery and in some cases hemorrhagic with marked hyperemia, ulceration, and hemorrhage of the intestinal mucosa.^2^ It has been proposed that an inflammatory process of the gastrointestinal tract could result in the release of hemoglobin carrying oxygen to the lumen of the colon and resulting in increased oxygen concentrations. An increase in oxygen concentrations in the mucosa and lumen of the intestine would benefit the proliferation of facultative anaerobes and a decrease in oxygen‐sensitive obligate anaerobes (i.e., Ruminococcaceae and Lachnospiraceae) the so‐called “oxygen hypothesis.”^43^, ^44^ Although this hypothesis is still under investigation, an increase in oxygen concentrations in the mucosa and lumen of the intestine could explain, at least in part, our findings. The production of short‐chain fatty acids (especially butyrate) by bacterial communities of the gastrointestinal tract maintains low oxygen and nitrate concentrations in the lumen of the gastrointestinal tract.^44^ Therefore, decreases in butyrate‐producing bacteria (eg, Lachnospiraceae, *Ruminococcus*, and *Faecalibacterium*) could have contributed to and facilitated proliferation of facultative anaerobes in horses with colitis. Luminal pH and nutrient availability (e.g., simple sugars) also play important roles in bacterial growth and metabolism.^46^, ^47^ Fecal pH and metabolite contents were not determined in our study, but it is likely that alteration in luminal pH and nutrient limitation during colonic inflammation contributed to the changes in bacterial communities during colitis. Alterations in gastrointestinal microbiota, especially the increased abundance of *Lactobacillus*, *Enterococcus*, and Enterobacteriaceae in horses with colitis, is of interest because proliferation of these taxa can enhance and perpetuate intestinal inflammation, mucosal damage, and facilitate translocation of bacteria and their by‐products.^43^, ^44^ Translocation of bacteria and their by‐products could explain some of the clinicopathological findings in the horses with colitis (e.g., tachycardia, fever, leukopenia).

Laminitis has been associated with an alteration of the gastrointestinal microbiota and absorption of microbial byproducts through permeable inflamed intestinal mucosa,^15^ but this association only has been established in experimental studies. Our study showed that the feces of horses with colitis that develop laminitis was enriched in *Lactobacillu*s, *Streptococcus*, and Enterobacteriaceae. Laminitis induced by exposure to high amounts of dietary carbohydrates (starch or oligofructose) also results in an increase in *Lactobacillus* and *Streptococcus*.^17^, ^18^, ^19^, ^20^ These changes correlated significantly with a decrease in cecal fluid pH and an increase in luminal lactate concentrations.^17^, ^18^, ^19^, ^20^ During the development of laminitis, Enterobacteriaceae populations initially increase but then decrease significantly.^15^ The decrease in Enterobacteriaceae is attributed to the reduction in cecal fluid pH, causing lysis of Enterobacteriaceae and consequent endotoxin release into the cecum. Along with an acidic colonic environment, these changes cause mucosal damage and facilitate translocation of bacteria, endotoxin, and bacterial by‐products (e.g., histamine, tryptamine, phenylethylamine, 5‐hydroxytryptamine).^48^, ^49^ These alterations are hypothesized to induce lamellar separation and consequent laminitis.^15^, ^49^ Our study was limited because fecal and blood metabolomic analyses were not available to determine correlation between bacteria and by‐products associated with the development of laminitis. These data could have helped confirm whether vasoactive amines and other bacterial by‐products produced in the lumen of the colon and absorbed into systemic circulation contribute to the development of laminitis in horses with colitis. The relative increased abundance of *Lactobacillus* in horses with colitis that develops laminitis raises the question of whether administering probiotics containing lactic acid‐producing bacteria could be harmful for horses with acute diarrhea.

Analysis of the fecal microbiota of surviving and nonsurviving horses with colitis, including horses that developed laminitis, indicated an enrichment of *Streptococcus* and *Lactobacillus* from the phylum Firmicutes. Critically ill humans with SIRS have higher total facultative anaerobe counts and lower total anaerobic bacterial counts compared with healthy individuals.^51^ The reasons for the association between changes in the gastrointestinal microbiota and mortality in the horses in our study is unknown, but could be related to the effect of the microbiota on the immune response.^50^ It is also possible that enrichment of facultative anaerobes (eg, *Lactobacillus* and *Streptococcus*) in nonsurviving horses indeed indicates a more severe inflammatory process in the gastrointestinal tract. These facilitate anaerobes in turn can result in more critical damage to the intestinal mucosa, decreased colonization resistance, and subsequent absorption of bacteria, toxins, and vasoactive amines,^30^, ^48^, ^49^, ^51^, ^52^ leading to an increased risk of mortality.^8^, ^12^ Removal of laminitis horses from the survival analysis confirmed that enrichment of *Streptococcus* was associated with nonsurvival of horses with colitis, suggesting a potential role of this bacterium in the severity of the intestinal inflammation and subsequent development of systemic clinical signs.^15^, ^16^, ^18^, ^19^, ^53^

The main limitation of our study was that the environmental and management practices of horses with colitis did not match those of the healthy group. Studies have shown that factors such as stress, diet, exercise, drugs, and disease may influence the gastrointestinal microbiota.^54^ However, the PCoA plots showed a differential clustering between healthy and colitis horses indicating that, despite differences in environment and management practices, healthy horses shared certain taxa that differentiated them from horses with colitis. Future studies including control farm horses are warranted to validate our results. Another limitation of our study was selection of a group of critically ill horses with colitis for the fecal microbiota analysis. The results could be biased toward horses with critical disease because severely affected horses are more likely to be referred to a tertiary hospital. Therefore, caution should be exercised in extrapolating our results to a different population. However, the horses admitted to our hospital are likely similar to those admitted to other tertiary hospitals. The approach used to define laminitis in the horses in our study is also a limitation. A horse with prodromic signs of laminitis or with laminitis but with radiographs that were normal or not performed would have been missed. The inclusion criteria aimed to eliminate subjectivity in the diagnosis of laminitis. An additional limitation of our study is that changes in housing, diet, or travel history before admission of horses with colitis were not consistently recorded. Changes in diet^55^ and transport^56^, ^57^ can alter the fecal microbiota of healthy horses, and such changes could have impacted the results of our study. Finally, most horses included in our study were not tested for all of the etiologic agents known to cause acute diarrhea in horses. This lack of testing prevented us from determining associations between specific pathogens and outcomes (e.g., laminitis and survival) and changes in the gastrointestinal microbiota.

In summary, we showed that dysbiosis occur in horses with colitis with an enrichment of facultative anaerobes and a decrease in some obligate anaerobic bacteria. Although we failed to detect differences in the community structure between laminitic and non‐laminitic horses or between surviving and nonsurviving horses with colitis, specific taxa were enriched in horses with colitis that developed laminitis or died. These findings suggest that the gastrointestinal microbiota could play an important role in the development of laminitis and the severity of disease in horses with colitis.

## CONFLICT OF INTEREST DECLARATION

Authors declare no conflict of interest.

## OFF‐LABEL ANTIMICROBIAL DECLARATION

Authors declare no off‐label use of antimicrobials.

## INSTITUTIONAL ANIMAL CARE AND USE COMMITTEE (IACUC) OR OTHER APPROVAL DECLARATION

Approved by University of Guelph Animal Care Committee (AUP#4540).

## HUMAN ETHICS APPROVAL DECLARATION

Authors declare human ethics approval was not needed for this study.

## Supporting information


**Figure S1** A. Relative abundance (median) of the more abundant genera identified in surviving (n = 20) and non‐surviving (n = 19) horses with colitis after removing horses that developed laminitis associated with colitis (n = 15) and one horse with chronic laminitis. B. Plot from LEfSe analysis indicating enriched taxa in fecal samples from surviving (n = 27) and non‐surviving (n = 28) horses with colitis after removing horses with laminitis. Linear discriminant analysis (LDA) cut off > 3.5 and P < .05. C. Principal coordinates analysis (PCoA) based on Jaccard index analysis of the bacterial 16S rRNA gene sequence data for fecal samples collected from surviving (n = 20) and non‐surviving (n = 19) horses with colitis after removing horses with laminitis. D. Principal coordinates analysis (PCoA) based on Yue and Clayton index analysis of the bacterial 16S rRNA gene sequence data for fecal samples collected from surviving (n = 20) and non‐surviving (n = 19) horses with colitis after removing horses with laminitis.

**Table S1** Alpha diversity comparisons of the Chao, Shannon Evenness, and Inverse Simpson's indices of healthy horses and horses with colitis.

**Table S2** Alpha diversity comparisons of the Chao, Shannon Evenness, and Inverse Simpson's indices of laminitis and non‐laminitis horses and surviving and nonsurviving horses

**Table S3** Relative abundance (median and range) of the most abundant taxa identified in healthy horses and diarrheic horses with or without laminitis

**Table S4** Relative abundance (median [%] and range) of the most abundant taxa identified in healthy horses and survival and nonsurviving diarrheic horses.

## References

[1] Shaw SD , Stämpfli H . Diagnosis and treatment of undifferentiated and infectious acute diarrhea in the adult horse. Vet Clin North Am Equine Pract. 2018;34:39‐53. 29426709 10.1016/j.cveq.2017.11.002 PMC7134835

[2] Uzal FA , Arroyo LG , Navarro MA , Gomez DE , Asin J , Henderson E . Bacterial and viral enterocolitis in horses: a review. J Vet Diag Investig. 2022;34(3):354‐375. 10.1177/10406387211057469 PMC9254067 34763560

[3] Gomez DE , Arroyo LG , Stämpfli HR , Cruz LE , Oliver OJ . Physicochemical interpretation of acid‐base abnormalities in 54 adult horses with acute severe colitis and diarrhea. J Vet Intern Med. 2013;27:548‐553. 23551698 10.1111/jvim.12071

[4] Costa MC , Arroyo LG , Allen‐Vercoe E , et al. Comparison of the fecal microbiota of healthy horses and horses with colitis by high throughput sequencing of the V3‐V5 region of the 16S rRNA gene. PLoS One. 2012;7(7):e41484. 22859989 10.1371/journal.pone.0041484 PMC3409227

[5] Arroyo LG , Rossi L , Santos BP , Gomez DE , Surette MG , Costa MC . Luminal and mucosal microbiota of the cecum and large colon of healthy and diarrheic horses. Animals (Basel). 2020;10(8):1403. 32806591 10.3390/ani10081403 PMC7460328

[6] Rodriguez C , Taminiau B , Brévers B , et al. Faecal microbiota characterisation of horses using 16 rdna barcoded pyrosequencing, and carriage rate of clostridium difficile at hospital admission. BMC Microbiol. 2015;16(15):181. 10.1186/s12866-015-0514-5 PMC4573688 26377067

[7] Berryhill EH , Magdesian KG , Aleman M , Pusterla N . Clinical presentation, diagnostic findings, and outcome of adult horses with equine coronavirus infection at a veterinary teaching hospital: 33 cases (2012‐2018). Vet J. 2019;248:95‐100. 31113572 10.1016/j.tvjl.2019.05.001 PMC7110482

[8] Cohen ND , Woods AM . Characteristics and risk factors for failure of horses with acute diarrhea to survive: 122 cases (1990‐1996). J Am Vet Med Assoc. 1999;214:382‐390. 10023402

[9] Magdesian KG , Dujowich M , Madigan JE , Hansen LM , Hirsh DC , Jang SS . Molecular characterization of *Clostridium difficile* isolates from horses in an intensive care unit and association of disease severity with strain type. J Am Vet Med Assoc. 2006;228(5):751‐755. 16506942 10.2460/javma.228.5.751

[10] Weese JS , Staempfli HR , Prescott JF . A prospective study of the roles of clostridium difficile and enterotoxigenic *Clostridium perfringens* in equine diarrhoea. Equine Vet J. 2001;33(4):403‐409. 11469775 10.2746/042516401776249534

[11] Parsons CS , Orsini JA , Krafty R , Capewell L , Boston R . Risk factors for development of acute laminitis in horses during hospitalization: 73 cases (1997‐2004). J Am Vet Med Assoc. 2007;230:885‐889. 17362165 10.2460/javma.230.6.885

[12] Groover ES , Woolums AR , Cole DJ , LeRoy BE . Risk factors associated with renal insufficiency in horses with primary gastrointestinal disease: 26 cases (2000‐2003). J Am Vet Med Assoc. 2006;228(4):572‐577. 16478436 10.2460/javma.228.4.572

[13] Elliott J , Bailey SR . Gastrointestinal derived factors are potential triggers for the development of acute equine laminitis. J Nutr. 2006;136(7 Suppl):2103‐2107. 10.1093/jn/136.7.2103S 16772511

[14] Luethy D , Feldman R , Stefanovski D , Aitken MR . Risk factors for laminitis and nonsurvival in acute colitis: retrospective study of 85 hospitalized horses (2011‐2019). J Vet Intern Med. 2020;35(4):1‐7. 10.1111/jvim.16147 PMC8295695 33938584

[15] Milinovich GJ , Klieve AV , Pollitt CC , Trott DJ . Microbial events in the hindgut during carbohydrate‐induced equine laminitis. Vet Clin North Equine Pract. 2010;26:79‐94. 10.1016/j.cveq.2010.01.007 20381737

[16] Bailey SR , Menzies‐Gow NJ , Marr CM , Elliott J . The effects of vasoactive amines found in the equine hindgut on digital blood flow in the normal horse. Equine Vet J. 2004;36:267‐272. 15147136 10.2746/0425164044877297

[17] Garner HE , Moore JN , Johnson JH , et al. Changes in the caecal flora associated with the onset of laminitis. Equine Vet J. 1978;10(4):249‐252. 738266 10.1111/j.2042-3306.1978.tb02273.x

[18] Milinovich GJ , Burrell PC , Pollitt CC , et al. Microbial ecology of the equine hindgut during oligofructose‐induced laminitis. ISME J. 2008;2(11):1089‐1100. 18580970 10.1038/ismej.2008.67

[19] Milinovich GJ , Trott DJ , Burrell PC , et al. Fluorescence in situ hybridization analysis of hindgut bacteria associated with the development of equine laminitis. Environ Microbiol. 2007;9(8):2090‐2100. 17635552 10.1111/j.1462-2920.2007.01327.x

[20] Tuniyazi M , He J , Guo J , et al. Changes of microbial and metabolome of the equine hindgut during oligofructose‐induced laminitis. BMC Vet Res. 2021;17(1):11. 33407409 10.1186/s12917-020-02686-9 PMC7789226

[21] Nicholson SE , Merrill D , Zhu C , et al. Polytrauma independent of therapeutic intervention alters the gastrointestinal microbiome. Am J Surg. 2018;216(4):699‐705. 30100050 10.1016/j.amjsurg.2018.07.026

[22] Houlden A , Goldrick M , Brough D , et al. Brain injury induces specific changes in the caecal microbiota of mice via altered autonomic activity and mucoprotein production. Brain Behav Immun. 2016;57:10‐20. 27060191 10.1016/j.bbi.2016.04.003 PMC5021180

[23] Huang G , Sun K , Yin S , et al. Burn injury leads to increase in relative abundance of opportunistic pathogens in the rat gastrointestinal microbiome. Front Microbiol. 2017;8:1237. 28729860 10.3389/fmicb.2017.01237 PMC5498482

[24] Nicholson SE , Burmeister DM , Johnson TR , et al. A prospective study in severely injured patients reveals an altered gut microbiome is associated with transfusion volume. J Trauma Acute Care Surg. 2019;86(4):573‐582. 30633104 10.1097/TA.0000000000002201 PMC6433524

[25] Waligora‐Dupriet AJ , Lafleur S , Charrueau C , et al. Head injury profoundly affects gut microbiota homeostasis: results of a pilot study. Nutrition. 2018;45:104‐107. 29129229 10.1016/j.nut.2017.06.026

[26] Kigerl KA , Hall JC , Wang L , et al. Gut dysbiosis impairs recovery after spinal cord injury. J Exp Med. 2016;213(12):2603‐2620. 27810921 10.1084/jem.20151345 PMC5110012

[27] Earley ZM , Akhtar S , Green SJ , et al. Burn injury alters the intestinal microbiome and increases gut permeability and bacterial translocation. PLoS One. 2015;10(7):e0129996. 26154283 10.1371/journal.pone.0129996 PMC4496078

[28] Burmeister DM , Johnson TR , Lai Z , et al. The gut microbiome distinguishes mortality in trauma patients upon admission to the emergency department. J Trauma Acute Care Surg. 2020;88(5):579‐587. 32039976 10.1097/TA.0000000000002612 PMC7905995

[29] Vaishnavi C . Translocation of gut flora and its role in sepsis. Indian J Med Microbiol. 2013;31:334‐342. 24064638 10.4103/0255-0857.118870

[30] Shimizu K , Ogura H , Hamasaki T , et al. Altered gut flora are associated with septic complications and death in critically ill patients with systemic inflammatory response syndrome. Dig Dis Sci. 2011;56(4):1171‐1177. 20931284 10.1007/s10620-010-1418-8 PMC3059822

[31] La Rosa PS , Brooks JP , Deych E , et al. Hypothesis testing and power calculations for taxonomic‐based human microbiome data. PLoS One. 2012;7(12):e52078. 23284876 10.1371/journal.pone.0052078 PMC3527355

[32] Klindworth A , Pruesse E , Schweer T , et al. Evaluation of general 16S ribosomal RNA gene PCR primers for classical and next‐generation sequencing‐based diversity studies. Nucleic Acids Res. 2013;41(1):e1. 22933715 10.1093/nar/gks808 PMC3592464

[33] Walters W , Hyde ER , Berg‐Lyons D , et al. Improved bacterial 16S rRNA gene (V4 and V4‐5) and fungal internal transcribed spacer marker gene primers for microbial community surveys. mSystems. 2015;1(1):e00009‐e00015. 27822518 10.1128/mSystems.00009-15 PMC5069754

[34] Schloss PD , Westcott SL , Ryabin T , et al. Introducing mothur: open‐source, platform‐independent, community‐supported software for describing and comparing microbial communities. Appl Environ Microbiol. 2009;75(23):7537‐7541. 19801464 10.1128/AEM.01541-09 PMC2786419

[35] Kozich JJ , Westcott SL , Baxter NT , Highlander SK , Schloss PD . Development of a dual‐index sequencing strategy and curation pipeline for analyzing amplicon sequence data on the MiSeq Illumina sequencing platform. Appl Environ Microbiol. 2013;79:5112‐5120. 23793624 10.1128/AEM.01043-13 PMC3753973

[36] Good IJ . The population frequencies of species and the estimation of population parameters. Biometrika. 1953;40:237‐264.

[37] Jaccard P . Nouvelles recherches sur la distribution florale. Nature. 1908;44:223‐270.

[38] Yue JC , Clayton MK . Similarity measure based on species proportions. Commun Stat Methods. 2005;34:2123‐2131.

[39] Holmes I , Harris K , Quince C . Dirichlet multinomial mixtures: generative models for microbial metagenomics. PLoS One. 2012;7:e30126. 22319561 10.1371/journal.pone.0030126 PMC3272020

[40] Benjamini Y , Hochberg Y . Controlling the false discovery rate: a practical and powerful approach to multiple testing. J R Stat Soc. 1995;57:289‐300.

[41] Segata N , Izard J , Waldron L , et al. Metagenomic biomarker discovery and explanation. Genome Biol. 2011;12(6):R60. 21702898 10.1186/gb-2011-12-6-r60 PMC3218848

[42] Pasolli E , Truong DT , Malik F , Waldron L , Segata N . Machine learning meta‐analysis of large metagenomic datasets: tools and biological insights. PLoS Comput Biol. 2016;12:1‐26. 10.1371/journal.pcbi.1004977 PMC4939962 27400279

[43] Rigottier‐Gois L . Dysbiosis in inflammatory bowel diseases: the oxygen hypothesis microbe‐microbe and microbe‐host interactions. ISME J. 2013;7:1256‐1261. 23677008 10.1038/ismej.2013.80 PMC3695303

[44] Stecher B . The roles of inflammation, nutrient availability and the commensal microbiota in enteric pathogen infection. Microbiol Spectrum. 2015;3:3. 10.1128/microbiolspec.MBP-0008-2014 26185088

[45] Shin NR , Whon TW , Bae JW . Proteobacteria: microbial signature of dysbiosis in gut microbiota. Trends Biotechnol. 2015;33:496‐503. 26210164 10.1016/j.tibtech.2015.06.011

[46] Ilhan ZE , Marcus AK , Kang DW , Rittmann BE , Krajmalnik‐Brown R . pH‐mediated microbial and metabolic interactions in fecal enrichment cultures. mSphere. 2017;2(3):e00047‐e00017. 28497116 10.1128/mSphere.00047-17 PMC5415631

[47] Sorbara MT , Pamer EG . Interbacterial mechanisms of colonization resistance and the strategies pathogens use to overcome them. Mucosal Immunol. 2019;12:1‐9. 29988120 10.1038/s41385-018-0053-0 PMC6312114

[48] Bailey SR , Baillon ML , Rycroft AN , Harris PA , Elliott J . Identification of equine cecal bacteria producing amines in an in vitro model of carbohydrate overload. Appl Environ Microbiol. 2003;69:2087‐2093. 12676687 10.1128/AEM.69.4.2087-2093.2003 PMC154823

[49] Bailey SR , Marr CM , Elliott J . Current research and theories on the pathogenesis of acute laminitis in the horse. Vet J. 2004;167:129‐142. 14975387 10.1016/S1090-0233(03)00120-5

[50] Peled JU , Gomes ALC , Devlin SM , et al. Microbiota as predictor of mortality in allogeneic hematopoietic‐cell transplantation. N Engl J Med. 2020;382(9):822‐834. 32101664 10.1056/NEJMoa1900623 PMC7534690

[51] Shimizu K , Ogura H , Goto M , et al. Altered gut flora and environment in patients with severe SIRS. J Trauma. 2006;60(1):126‐133. 16456446 10.1097/01.ta.0000197374.99755.fe

[52] Yang XJ , Liu D , Ren HY , Zhang XY , Zhang J , Yang XJ . Effects of sepsis and its treatment measures on intestinal flora structure in critical care patients. World J Gastroenterol. 2021;27(19):2376‐2393. 34040329 10.3748/wjg.v27.i19.2376 PMC8130038

[53] Milinovich GJ , Burrell PC , Pollitt CC , Bouvet A , Trott DJ . *Streptococcus henryi* sp. nov. and *Streptococcus caballi* sp. nov., isolated from the hindgut of horses with oligofructose‐induced laminitis. Int J Syst Evol Microbiol. 2008;2008(58):262‐266. 10.1099/ijs.0.65063-0 18175719

[54] Garber A , Hastie P , Murray JA . Factors influencing equine gut microbiota: current knowledge. J Equine Vet Sci. 2020;88:102943. 32303307 10.1016/j.jevs.2020.102943

[55] Collinet A , Grimm P , Julliand S , Julliand V . Sequential modulation of the equine fecal microbiota and fibrolytic capacity following two consecutive abrupt dietary changes and bacterial supplementation. Animals (Basel). 2021;11(5):1278. 33946811 10.3390/ani11051278 PMC8144951

[56] Szemplinski KL , Thompson A , Cherry N , et al. Transporting and exercising unconditioned horses: effects on microflora populations. J Equine Vet Sci. 2020;90:102988. 32534767 10.1016/j.jevs.2020.102988

[57] Schoster A , Mosing M , Jalali M , Staempfli HR , Weese JS . Effects of transport, fasting and anaesthesia on the faecal microbiota of healthy adult horses. Equine Vet J. 2016;48(5):595‐602. 26122549 10.1111/evj.12479

