# Supplementary figures and images for: Fecal microbiota of horses with colitis and its association with laminitis and survival during hospitalization
Source: J Vet Intern Med. 2022 Oct 21;36(6):2213–23. doi: 10.1111/jvim.16562 (PMC9708523; doi:10.1111/jvim.16562)

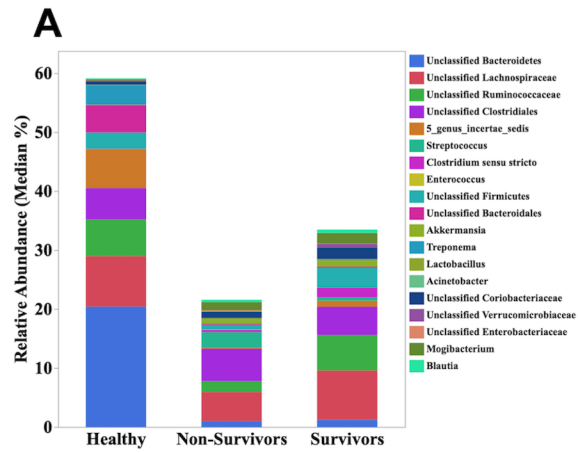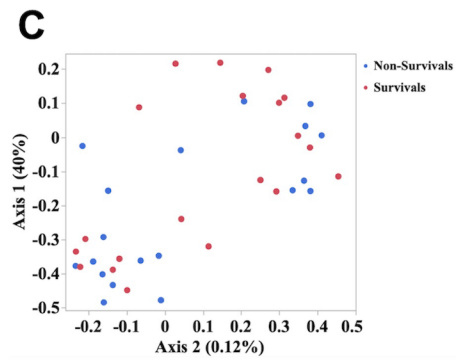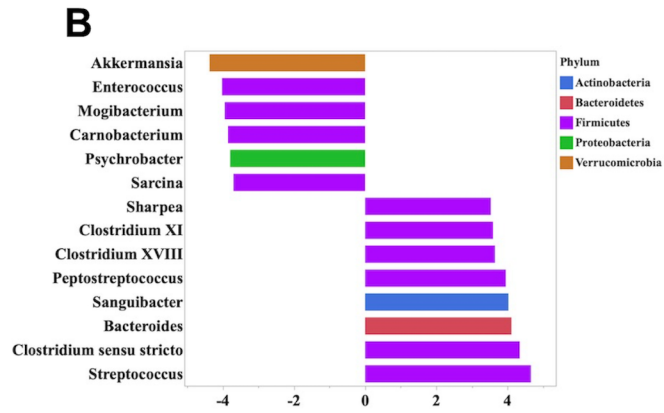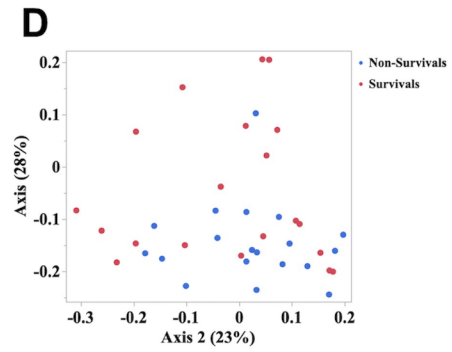

Supplement: Supplementary file 1 — Figure S1 A. Relative abundance (median) of the more abundant genera identified in surviving (n = 20) and non‐surviving (n = 19) horses with colitis after removing horses that developed laminitis associated with colitis (n = 15) and one horse with chronic laminitis. B. Plot from LEfSe analysis indicating enriched taxa in fecal samples from surviving (n = 27) and non‐surviving (n = 28) horses with colitis after removing horses with laminitis. Linear discriminant analysis (LDA) cut off > 3.5 and P < .05. C. Principal coordinates analysis (PCoA) based on Jaccard index analysis of the bacterial 16S rRNA gene sequence data for fecal samples collected from surviving (n = 20) and non‐surviving (n = 19) horses with colitis after removing horses with laminitis. D. Principal coordinates analysis (PCoA) based on Yue and Clayton index analysis of the bacterial 16S rRNA gene sequence data for fecal samples collected from surviving (n = 20) and non‐surviving (n = 19) horses with colitis after removing horses with laminitis. [file JVIM-36-2213-s004.pdf]
