# Supplementary material for: Fecal microbiota of horses with colitis and its association with laminitis and survival during hospitalization
Source: J Vet Intern Med. 2022 Oct 21;36(6):2213–23. doi: 10.1111/jvim.16562 (PMC9708523; doi:10.1111/jvim.16562)
Supplement: Supplementary file 2 — Table S1 Alpha diversity comparisons of the Chao, Shannon Evenness, and Inverse Simpson's indices of healthy horses and horses with colitis. [file JVIM-36-2213-s005.pdf]

Supplementary Table 1: Alpha diversity comparisons of the Chao, Shannon Evenness, and Inverse Simpson's indices of healthy horses and horses with colitis.

**Index**	**Healthy N = 36**	**Colitis N = 55**	**P- values**
**Chao-1 (richness)**	167 [129 – 241]	180 [108 – 344]	0.11
**Shannon Evenness (evenness)**	0.6 [0.5 – 0.6]	0.6 [0.07 – 0.6]	0.86
**Inverse Simpson (diversity)**	9 [6 – 17]	9 [1 – 16]	0.72

P-values were obtained with the Wilcoxon test. Median and range were included for each group.
