# Supplementary material for: Fecal microbiota of horses with colitis and its association with laminitis and survival during hospitalization
Source: J Vet Intern Med. 2022 Oct 21;36(6):2213–23. doi: 10.1111/jvim.16562 (PMC9708523; doi:10.1111/jvim.16562)
Supplement: Supplementary file 3 — Table S2 Alpha diversity comparisons of the Chao, Shannon Evenness, and Inverse Simpson's indices of laminitis and non‐laminitis horses and surviving and nonsurviving horses [file JVIM-36-2213-s001.pdf]

Supplementary Table 2. Alpha diversity comparisons of the Chao, Shannon Evenness, and Inverse Simpson's indices of laminitis and non-laminitis horses and surviving and non-surviving horses.

**Index**	**Laminitis N = 15**	**Non- laminitis N = 39**	**Survivors N = 27**	**Non-survivors N = 28**
**Chao-1 (richness)**	180 [125-344]	175 [108-319]	187 [108-319]	172 [111-344]
**Shannon Evenness (evenness)**	0.5 [0.4-0.6]	0.6 [0.07-0.6]	0.6 [0.07-0.6]	0.5 [0.3-0.6]
**Inverse Simpson (diversity)**	8 [3-13]	10 [1-16]	10 [1-16]	8 [2-16]

P-values were obtained with the Wilcoxon test. * Represents statistical difference between groups (p = .049*). Median and range were included for each group. One horse with chronic laminitis was removed from the laminitis analysis.
