# Supplementary material for: Fecal microbiota of horses with colitis and its association with laminitis and survival during hospitalization
Source: J Vet Intern Med. 2022 Oct 21;36(6):2213–23. doi: 10.1111/jvim.16562 (PMC9708523; doi:10.1111/jvim.16562)
Supplement: Supplementary file 4 — Table S3 Relative abundance (median and range) of the most abundant taxa identified in healthy horses and diarrheic horses with or without laminitis [file JVIM-36-2213-s003.pdf]

Supplementary Table 3. Relative abundance (median and range) of the most abundant taxa identified in healthy horses and diarrheic horses with or without laminitis

**Phylum**	**Family**	**Genus**	**Healthy N = 36**	**Laminitis N = 15**	**Non-Laminitis N =39**
Bacteroidetes	Unclass.	Unclass.	20.5^{a} [12.8 – 35.5]	0.68^{b} [0.025 – 9.0]	1.2^{b} [0.011 – 15.9]
Firmicutes	Lachnospiraceae	Unclass.	8.7^{a} [1.5 – 20.8]	2.7^{a} [0.19 – 18.5]	7.1^{a} [0.023 – 32.2]
Firmicutes	Ruminococcaceae	Unclass.	6.8^{a} [1.4 – 12.5]	1.6^{b} [0.21 – 13.7]	3.6^{ab} [0.021 – 22.8]
Firmicutes	Clostridiales Unclass.	Unclass.	5.4^{a} [0.75 – 8.4]	4.3^{a} [0.90 – 21.2]	5.1^{a} [0.014 – 14.0]
Verrucomocrobia	5_family_incertae_sedis	*5_genus_incertae_sedis*	6.7^{a} [1.7 – 16.5]	0.012^{b} [0.0051 – 5.9]	0.46^{b} [0.0041 – 9.5]
Firmicutes	Streptococcaceae	*Streptococcus*	0.026^{a} [0 – 7.5]	2.4^{b} [0.011 – 54.1]	1.0^{b} [0.0059 – 52.6]
Firmicutes	Clostridiaceae_1	*Clostridium_sensu_stricto*	0.058^{a} [0.00090 – 1.6]	0.18^{ab} [0.021 – 38.1]	1.0^{b} [0.00080 – 66.4]
FIrmicutes	Enterococcaceae	*Enterococcus*	0.0014^{a} [0 – 0.026]	0.4^{b} [0.0066 – 7.6]	0.061^{b} [0.0017 – 94.5]
Firmicutes	Unclass.	Unclass.	2.8^{a} [0.43 – 5.8]	1.7^{a} [0.095 – 7.8]	1.9^{a} [0.012 – 8.4]
Bacteroidetes	Bacteroidales Unclass.	Unclass.	4.7^{a} [0.49 – 6.9]	0.11^{b} [0.0043 – 0.87]	0.19^{b} [0.0023 – 14.0]
Verrucomicrobia	Verrucomicrobiaceae	*Akkermansia*	0.061^{a} [0.0026 – 1.8]	0.34^{ab} [0.0085 – 31.8]	0.76^{b} [0.00070 – 46.3]
Spirochaetes	Spirochaetaceae	*Treponema*	3.4^{a} [1.1 – 7.9]	0.024^{b} [0.0064 – 1.1]	0.091^{b} [0.00070 – 2.9]
Firmicutes	Lactobacillaceae	*Lactobacillus*	0.069^{a} [0.0034 – 1.1]	0.60^{a} [0.0068 – 33.2]	0.21^{a} [0.0051 – 33.3]
Proteobacteria	Moraxellaceae	*Acinetobacter*	0.016^{a} [0.0006 – 8.8]	0.0071^{a} [0 – 27.9]	0.020^{a} [0 – 40.3]
Actinobacteria	Coriobacteriaceae	Unclass.	0.45^{a} [0.040 – 4.2]	1.1^{a} [0.025 – 9.5]	1.4^{a}[0.0014 – 17.4]
Verrucomicrobia	Verrucomicrobiaceae	Unclass.	0.17^{a} [0.0079 – 3.0]	0.028^{a} [0.0011 – 18.4]	0.22^{a} [0.0011 – 23.6]
Proteobacteria	Enterobacteriaceae	Unclass.	0.0051^{a} [0 – 0.14]	0.17^{b} [0.0082 – 18.8]	0.091^{b} [0 – 38.7]
Firmciutes	Eubacteriaceae	*Mogibacterium*	0.21^{a} [0.020 – 2.0]	0.58^{ab} [0.0085 – 12.0]	1.5^{b} [0.0022 – 14.6]
Firmicutes	Lachnospiraceae	*Blautia*	0.16^{a} [0.013 – 2.2]	0.72^{a} [0.014 – 46.0]	0.50^{a} [0.0028 – 5.4]

Unclass, Unclassified taxonomy from the from the earlier identified taxonomic group. P-values obtained using Steel Dwass test for multiple comparisons and corrected with Benjamini and Hochberg's false discovery rate analysis (FDR). P-values with a FDR adjustment of < .05 were considered significant. Superscript letters indicate withing row comparisons that are significantly different.
