# Supplementary material for: Fecal microbiota of horses with colitis and its association with laminitis and survival during hospitalization
Source: J Vet Intern Med. 2022 Oct 21;36(6):2213–23. doi: 10.1111/jvim.16562 (PMC9708523; doi:10.1111/jvim.16562)
Supplement: Supplementary file 5 — Table S4 Relative abundance (median [%] and range) of the most abundant taxa identified in healthy horses and survival and nonsurviving diarrheic horses. [file JVIM-36-2213-s002.pdf]

Supplementary Table 4. Relative abundance (median (%) and range) of the most abundant taxa identified in healthy horses and survival and non-surviving diarrheic horses.

**Phylum**	**Family**	**Genus**	**Healthy**<br>**n = 36**	**Survivors**<br>**n = 27**	**Non-Survivors**<br>**n = 28**
Bacteroidetes	Unclass.	Unclass.	20.5^{a} [12.8 – 35.5]	1.4^{b} [0.014 – 15.9]	0.94^{b} [0.011 – 14.0]
Firmicutes	Lachnospiraceae	Unclass.	8.7^{a} [1.5 – 20.8]	5.2^{a} [0.43 – 18.5]	4.9^{a} [0.023 – 32.2]
Firmicutes	Ruminococcaceae	Unclass.	6.8^{a} [1.4 – 12.5]	3.6^{a} [0.22 – 20.5]	1.6^{a} [0.021 – 22.8]
Firmicutes	Clostridiales Unclass.	Unclass.	5.4^{a} [0.75 – 8.4]	5.3^{a} [0.023 – 21.2]	4.9^{a} [0.014 – 14.0]
Verrucomicrobia	5_family_incertae_sedis	*5_genus_incertae_sedis*	6.7^{a} [1.7 – 16.5]	1.0^{b} [0.0041 – 9.5]	0.15^{b} [0.0051 – 4.2]
Firmicutes	Streptococcaceae	*Streptococcus*	0.026^{a} [0 – 7.5]	0.64^{b} [0.01 – 34.7]	1.9^{b} [0.0059 – 54.1]
Firmicutes	Clostridiaceae_1	*Clostridium_sensu_stricto*	0.058^{a} [0.00090 – 1.6]	1.8^{b} [0.0008 – 66.4]	0.37^{b} [0.0032 – 43.5]
Firmciutes	Enterococcaceae	*Enterococcus*	0.0014^{a} [0 – 0.026]	0.18^{b} [0.0017 – 17.4]	0.30^{b} [0.0048 – 94.5]
Firmicutes	Unclass.	Unclass.	2.8^{a} [0.43 – 5.8]	2.0^{a} [0.015 – 7.8]	1.5^{a} [0.012 – 8.4]
Bacteroidetes	Bacteroidales Unclass	Unclass.	4.7^{a} [0.49 – 6.9]	0.20^{b} [0.0077 – 14.0]	0.18^{b} [0.0023 – 1.9]
Verrucomicrobia	Verrucomicrobiaceae	*Akkermansia*	0.061^{a} [0.0026 – 1.8]	0.65^{ab} [0.0085 – 26.5]	0.65^{b} [0.0007 – 46.2]
Spirochaetes	Spirochaetaceae	*Treponema*	3.4^{a} [1.1 – 7.9]	0.12^{b} [0.0008 – 2.9]	0.055 ^{b} [0.0007 – 2]
Firmicutes	Lactobacillaceae	*Lactobacillus*	0.069 ^{a} [0.0034 – 1.1]	0.15^{b} [0.0063 – 33.3]	0.50^{ab} [0.0051 – 9.4]
Proteobacteria	Moraxellaceae	*Acinetobacter*	0.016^{a} [0.0006 – 8.8]	0.025^{a} [0.0007 – 40.3]	0.011^{a} [0 – 27.8]
Actinobacteria	Coriobacteriaceae	Unclass.	0.45^{a} [0.040 – 4.2]	1.7^{ab} [0.0048 – 9.5]	1.2^{b} [0.0014 – 17.4]
Verrucomicrobia	Unclass.	Unclass.	0.14^{a} [0.013 – 0.37]	0.0039^{a} [0 – 0.27]	0.0006^{a} [0 – 0.12]
Proteobacteria	Enterobacteriaceae	Unclass.	0.0051^{a} [0 – 0.14]	0.085^{b} [0 – 38.7]	0.17^{b} [0.0046 – 18.8]
Firmicutes	Eubacteriaceae	*Mogibacterium*	0.21^{a} [0.020 – 2.0]	1.8^{ab} [0.0033 – 12.0]	0.68^{b} [0.0022 – 14.6]
Firmicutes	Lachnospiraceae	*Blautia*	0.16^{a} [0.013 – 2.2]	1.0^{a} [0.0071 – 46.0]	0.23^{a} [0.0028 – 4.7]

Unclass, Unclassified taxonomy from the from the earlier identified taxonomic group. P-values obtained using Steel Dwass test for multiple comparisons and corrected with Benjamini and Hochberg's false discovery rate analysis (FDR). P-values with a FDR adjustment of $< .05$ were considered significant. Superscript letters indicate withing row comparisons that are significantly different.
